# Tuberculosis in an Immunocompetent Immigrant Patient

**DOI:** 10.7759/cureus.10225

**Published:** 2020-09-03

**Authors:** Andrew Mekaiel, Amna Al-Tkrit, Mohammad Aneeb, Meena Saeed, Kaushik Doshi

**Affiliations:** 1 Internal Medicine, Jamaica Hospital Medical Center, Jamaica, USA

**Keywords:** peritoneal tuberculosis (tb), abdominal tuberculosis, gastrointestinal tuberculosis, immigrant, immunocompetent, anti-tuberculosis therapy, ascites, laparoscopy with peritoneal biopsy

## Abstract

Peritoneal tuberculosis (TB) is a rare medical condition in developed nations like the United States, and it is uncommon to observe this condition in patients without underlying immunosuppression. This report describes a patient who developed abdominal pain, constipation, and ascites. And later on, he was diagnosed with peritoneal TB following laparoscopy with peritoneal biopsy. The patient was an immigrant from a high TB burden country but had no other common risk factors for the development of peritoneal TB. Treatment with anti-TB therapy resulted in significant clinical improvement.

## Introduction

Peritoneal tuberculosis (TB) is a rarely occurring medical condition in Western countries. It is characterized by peritoneal infection by Mycobacterium tuberculosis via hematogenous or lymphatic spread from a primary lung focus, or through contiguous spread from an adjacent infected focus. In most cases, peritoneal TB develops in patients with underlying immunocompromised states, such as HIV infection. However, it may be seen without the presence of immunosuppression, such as in immigrants from areas endemic for TB [1]. In this report, we present a case of peritoneal TB in an otherwise immunocompetent patient who emigrated to the United States from Bangladesh and presented with abdominal pain, constipation, and new-onset lymphocytic ascites.

## Case presentation

A 72-year-old man from Bangladesh, who immigrated to the United States four years ago, presented to our hospital's emergency department with complaints of constipation and progressively worsening abdominal pain and distension for 10 days. The patient had no significant past medical history and reported no alcohol use. There was no recent travel history, and the patient denied any prior contact with sick patients or any known TB exposure.

His vital signs were within normal limits in the emergency room, with a temperature of 97.7˚F, blood pressure of 130/86 mmHg, heart rate of 71 beats/min, and respiratory rate of 16 breaths/min. On physical examination, the patient appeared frail and had a tense, distended abdomen with marked ascites. The cardiopulmonary examination was unremarkable. A complete blood count revealed a white blood cell (WBC) count of 4,400 cells/µL, a hemoglobin level of 13.7 g/dL, a hematocrit of 41.1%, and a platelet count of 485,000/µL. Serum chemistry findings were within normal limits as it showed blood urea nitrogen (BUN) 12 mg/dL (7-20 mg/dL), creatinine 0.8 mg/dL (0.8-1.2 mg/dL), sodium 136 mEq/L (135-145 mEq/L), potassium 3.8 mEq/L (3.6-5.2 mEq/L), chloride 100 mEq/L (97-107 mEq/dL), and calcium 8.9 mg/dL (8.5-10.5 mg/dL). Liver function tests showed an alanine aminotransferase (ALT) level of 34 U/L (29-34 U/L) and an aspartate aminotransferase (AST) level of 26 U/L (5-40 U/L). Viral hepatitis and QuantiFERON®-TB tests were found to be negative. The carcinoembryonic antigen (CEA) level was 2.27 ng/mL (reference 0-3 ng/mL). An elevated cancer antigen-125 (CA-125) level of 307 units/mL (reference 0-35 units/mL) was noted to be present.

**Figure 1:**
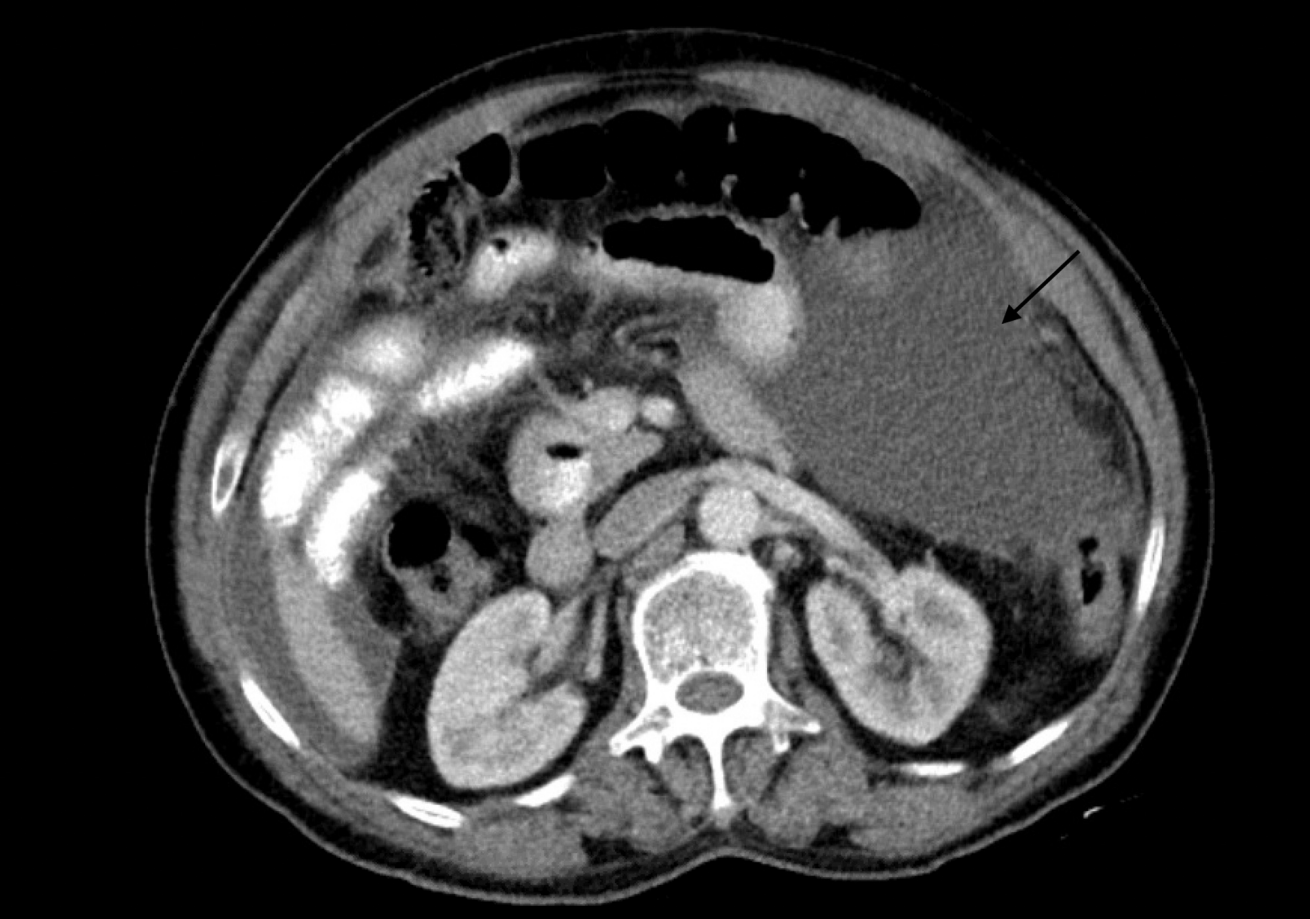
CT scan of the abdomen and pelvis revealing abdominal ascites (arrow)

CT scan of the abdomen and pelvis with contrast showed abdominal and pelvic ascites (Figure 1). Ultrasound-guided paracentesis was performed, and approximately 550 mL of yellowish fluid was obtained. The ascitic fluid analysis revealed a total red blood cell count of 250 cells/µL and a total WBC count of 1,065 cells/µL, with 98% lymphocytes. Cytology was negative for malignant cells as well as for acid-fast bacilli (AFB). The peritoneal fluid adenosine deaminase (ADA) level was 40 U/L (reference < 9.2 U/L). The gastroenterology team recommended upper gastrointestinal (GI) endoscopy and colonoscopy to rule out a GI malignancy. The GI workup was negative for any significant pathology. During his hospital stay, the patient developed worsening dyspnea, and a right-sided pleural effusion was noted to be present. CT-guided pleural drainage with pleural biopsy was performed and yielded non-significant inflammatory cells without any malignant cells.

**Figure 2:**
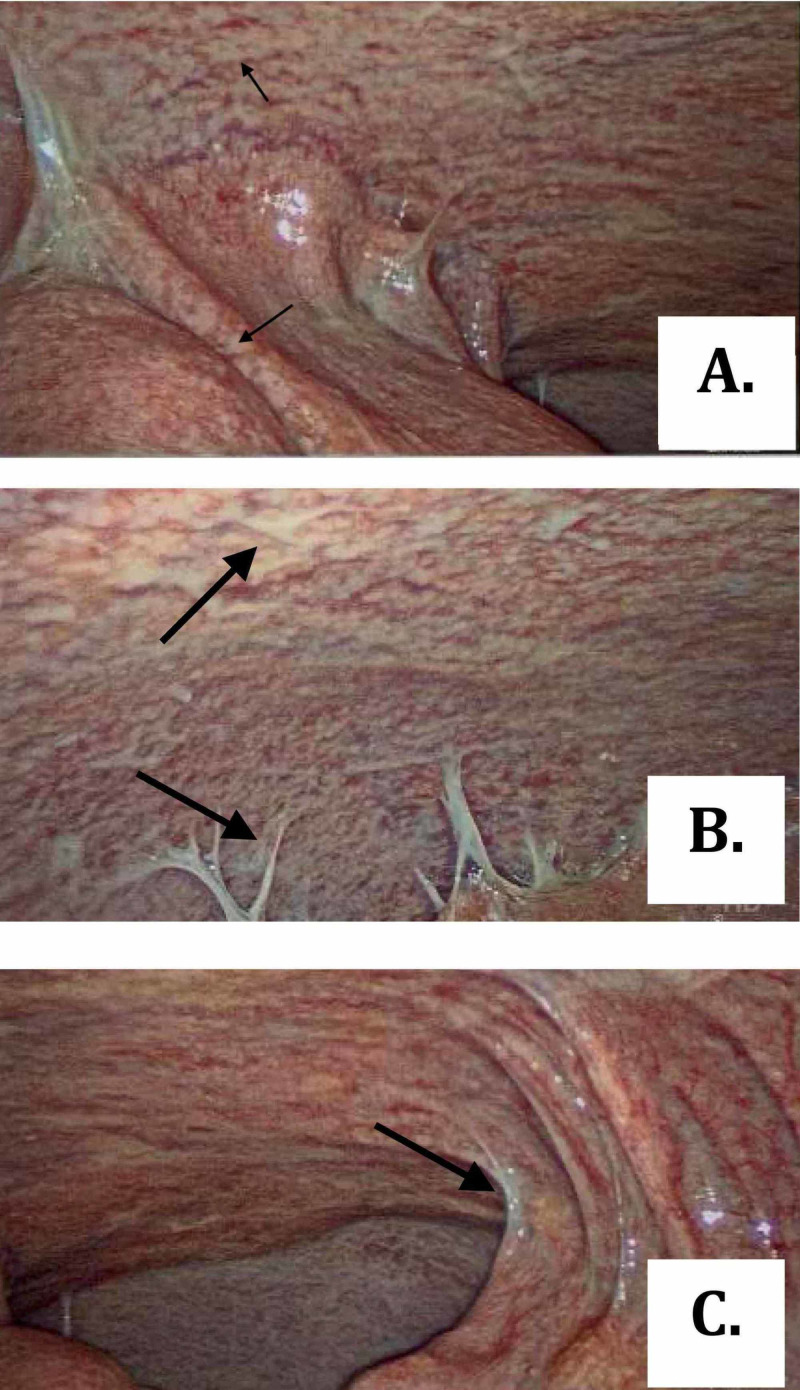
(A-C) Laparoscopic images showing multiple miliary white plaques over peritoneal surfaces (arrows)

**Figure 3:**
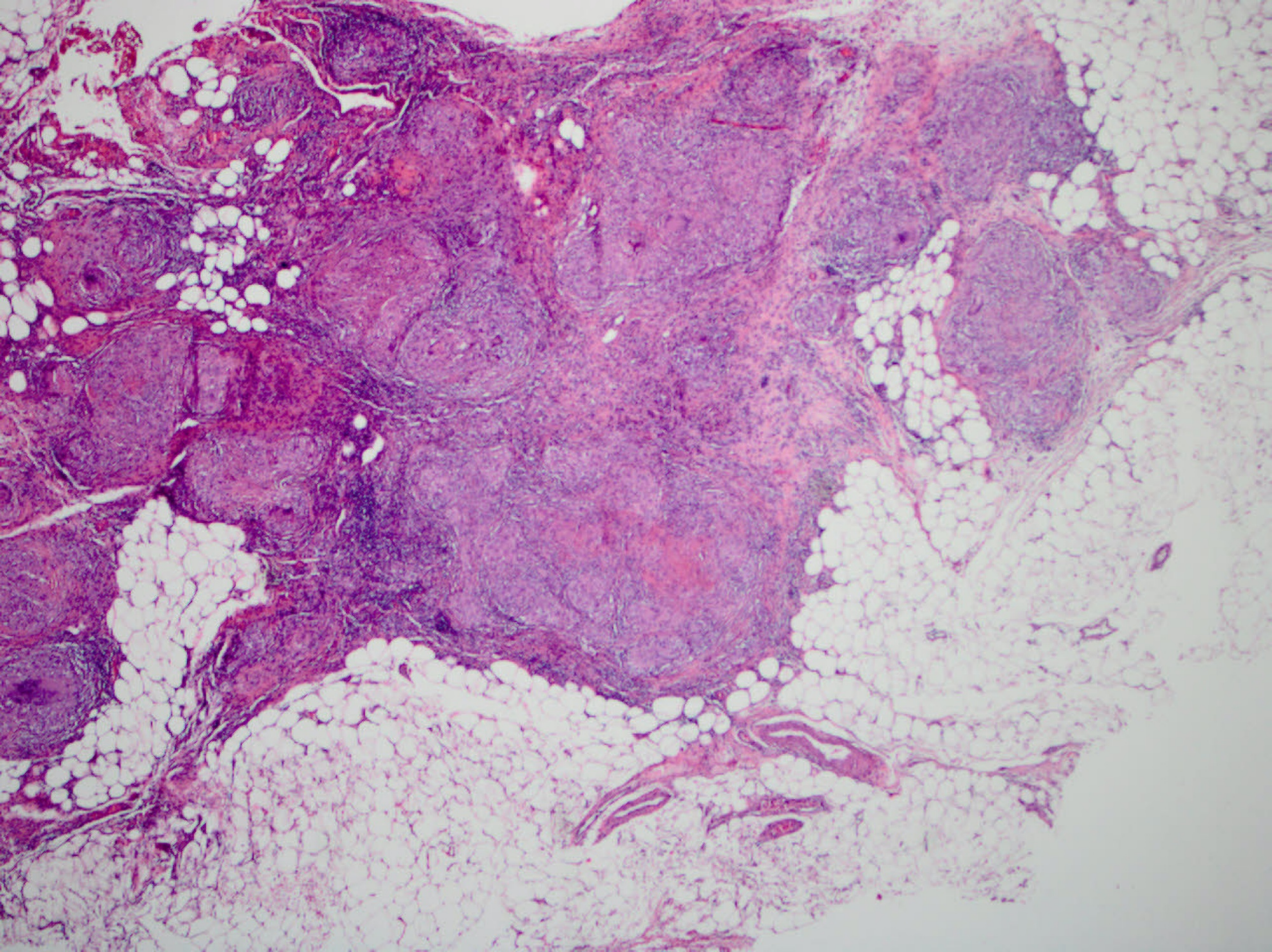
Microscopic image of peritoneal adipose tissue partly occupied by confluent granulomas (×40, hematoxylin & eosin stain)

**Figure 4:**
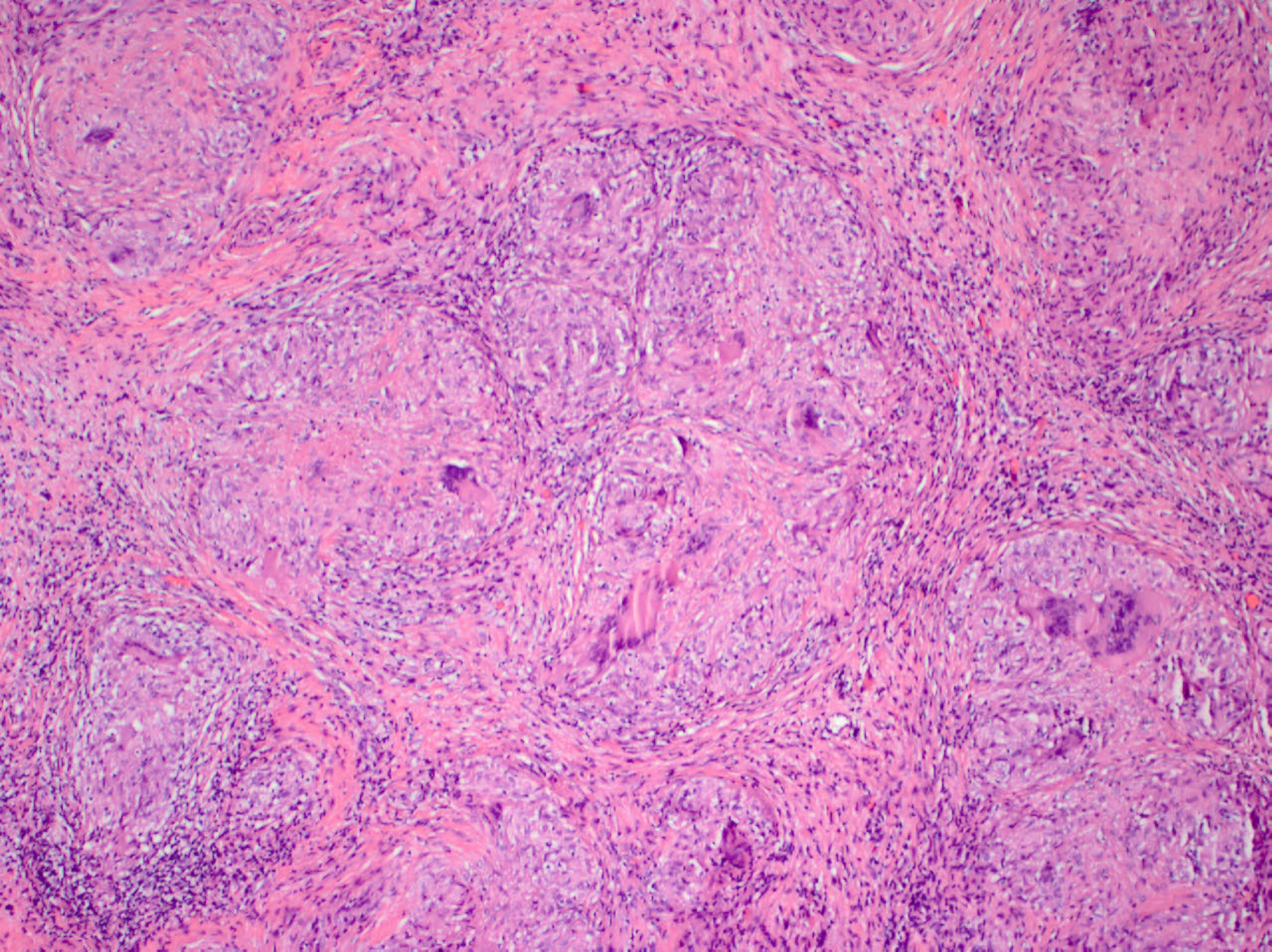
Microscopic image exhibitng conflent granulomas, focally necrotizing (×100, hematoxylin & eosin stain)

**Figure 5:**
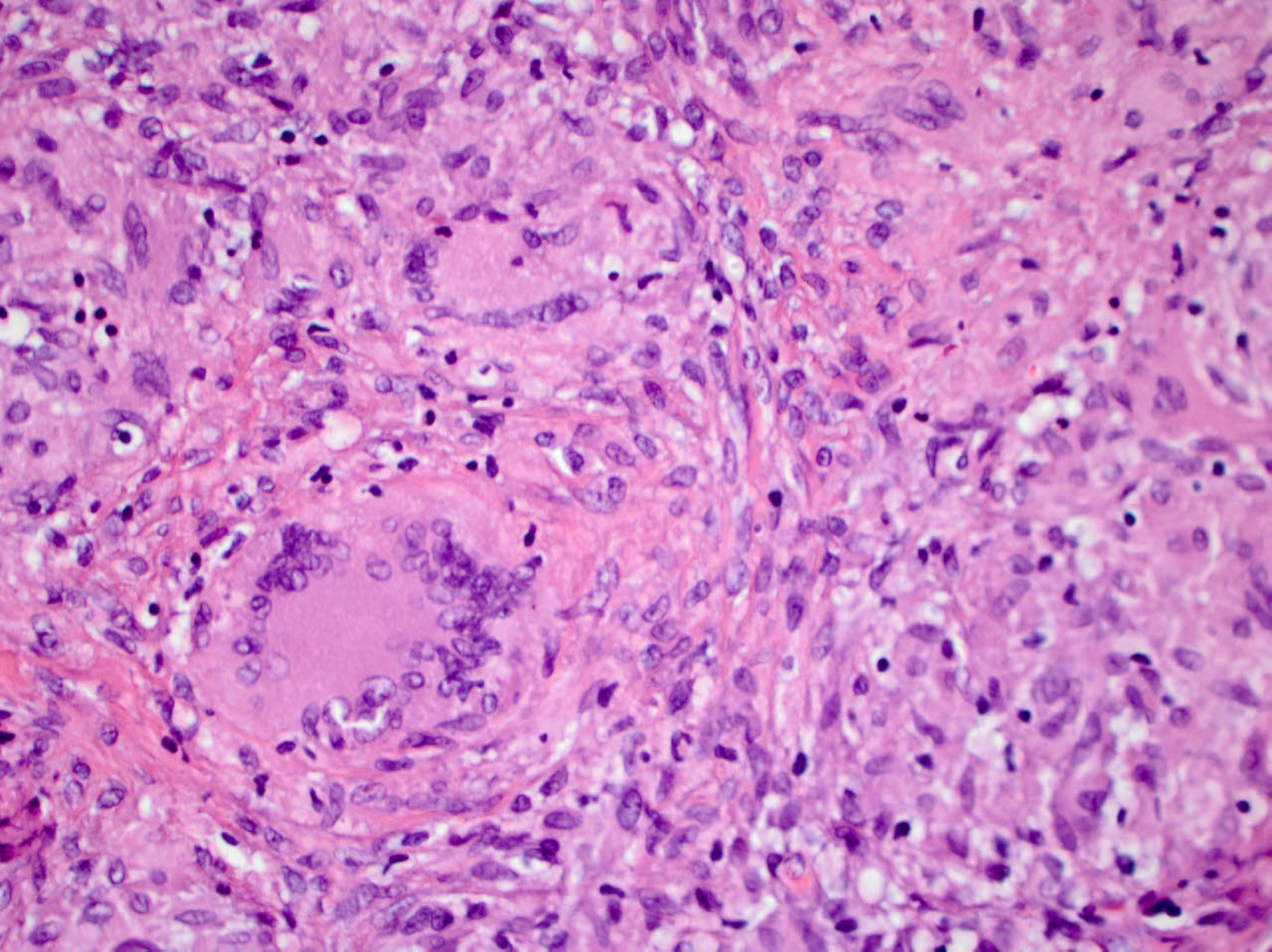
Microscopic image of confluent granulomata showing multinucleated giant cells (×400, hematoxylin & eosin stain)

Provided the high suspicion of peritoneal TB, we consulted general surgery, and the patient underwent laparoscopy with peritoneal biopsy. Laparoscopy revealed diffuse, miliary white plaques along all peritoneal and visceral surfaces (Figure 2). Peritoneal biopsy showed granulomas with focal necrosis, and AFB staining revealed acid-fast bacteria, thus confirming the diagnosis of peritoneal TB (Figures 3-5). The infectious disease department recommended initiating anti-TB treatment. The patient was discharged from the hospital on four anti-TB medications: rifampin 600 mg daily, isoniazid 300 mg daily, pyrazinamide 1,500 mg daily, and ethambutol 1,200 mg daily. The total hospital length of stay was 25 days. A two-month follow-up showed significant symptomatic improvement with the resolution of ascites and abdominal distension.

## Discussion

Abdominal TB is a significant health problem in developing countries but is an uncommon condition in the Western world. However, the rate of infection in developed countries appears to be rising. This may partly be because of the increase in travel and migration, and also because of the increasing numbers of HIV patients who are prone to contract opportunistic infections. Abdominal TB typically manifests as four different forms: tuberculous lymphadenopathy, GI TB, visceral TB, and peritoneal TB. Occasional overlaps between these four forms may be present in any individual patient. The peritoneum is considered the sixth most common extrapulmonary site for TB infection in the United States. Peritoneal TB accounts for almost 31%-58% of cases of abdominal TB and may be seen in around 3.5% of cases of pulmonary TB [2]. Immunocompromised states, such as HIV infection, liver cirrhosis, systemic steroid use, advanced renal failure requiring continuous ambulatory peritoneal dialysis (CAPD), and underlying malignancy, have been associated with an increased risk for the development of peritoneal TB in most cases [2,3]. It is important to note that, in contrast to the native-born individuals in the Western countries, immigrants from TB-endemic areas mostly develop extrapulmonary TB in the absence of any identifiable immunosuppression [4].

Peritoneal TB may be divided into three types: wet-ascitic, fibrotic-fixed, and dry plastic. The wet-ascitic type is defined by large amounts of loculated or free, high-protein ascitic fluid. It is the more common of the three types. The fibrotic-fixed type is characterized by adhesions of the bowel along with the omentum and mesentery. Loculated ascites may occasionally be present. A gross inflammatory reaction demonstrated by the formation of diffuse fibrous peritoneal adhesions and nodules is seen in the dry plastic type. Peritoneal TB usually presents as a combination of these three variants [5,6]. The pathologic mechanism involves the peritoneum infection by Mycobacterium tuberculosis via three main pathways. Most commonly, the tubercle bacilli infect the peritoneum following the reactivation of TB in the lungs and the subsequent hematogenous or lymphatic spread from a primary lung focus. The same mechanism may also lead to the development of peritoneal TB in patients with active pulmonary or miliary TB. Less commonly, peritoneal involvement may occur following the ingestion of infected ingested milk. The mucosal layer of the GI tract becomes infected, and epithelioid tubercles are subsequently formed in the lymphoid tissue of the submucosa. These tubercles undergo caseous necrosis in about two to four weeks, resulting in mucosal ulceration and infection of the intestines' deeper layers. The infection can then spread to the adjacent lymph nodes and peritoneum. Finally, peritoneal TB may develop due to the direct spread of infection from an infected adjacent focus, such as a psoas abscess or fallopian tubes in women [1,7].

The clinical manifestations of peritoneal TB are nonspecific and frequently overlap with other abdominal conditions, such as inflammatory bowel disease, liver cirrhosis with ascites, and spontaneous bacterial peritonitis. The initial presentation may also be concerning for malignancies, for instance, peritoneal carcinomatosis or advanced ovarian carcinoma. A significant variability may be present in symptom onset, and the duration of illness may range from several weeks to months. Most patients with peritoneal TB present with vague abdominal pain, fever, diarrhea and constipation, abdominal distension, anorexia, malaise, and weight loss. Physical examination may reveal diffuse abdominal tenderness, doughy abdomen, and abdominal ascites. A palpable abdominal mass, indicating the presence of matted bowel loops, may also be present [7-9].

Due to the lack of specific signs and symptoms and the limited yield of the commonly used diagnostic investigations, peritoneal TB diagnosis poses a significant challenge to the clinicians. Routine hematologic indices are usually nonspecific, and are thus of low yield. Interferon-gamma release assays (IGRAs), such as the QuantiFERON-TB gold and T-spot TB tests, assess interferon-gamma levels released by the T-lymphocytes when exposed to synthetic TB antigen. However, the frequently immunocompromised states of patients with peritoneal TB most likely lead to indeterminate results. Ascitic fluid analysis usually shows an elevated WBC count, ranging from 500 to 1,500 cells/µL, with lymphocytic predominance in most patients. High protein levels (>2.5 mg/dL) and serum-ascites albumin gradient (SAAG) less than 1.1 g/dL are also typically noticed. AFB staining and Ziehl-Neelsen (ZN) staining of the ascitic fluid are positive in only 3% of the cases, whereas bacterial cultures may be positive in 21%-35% of patients. Ascitic ADA levels ≥ 30 U/L have been shown to have a high sensitivity for the diagnosis of peritoneal TB [2]. CA-125 is a nonspecific tumor marker for ovarian carcinoma and has also been reported to be elevated in peritoneal TB [8]. Imaging studies, such as abdominal ultrasound and CT, also demonstrate nonspecific findings, including ascites with fine septations, lymphadenopathy, and thickening of the peritoneum, mesentery, or omentum [1]. Laparoscopy with peritoneal biopsy and subsequent microbiologic confirmation remains the gold standard for establishing peritoneal TB diagnosis. The typical laparoscopic findings include ascites, thickened peritoneum, adhesions, and whitish miliary nodules scattered over the peritoneum. The histological analysis of the peritoneal biopsies most commonly reveals granulomas with caseation necrosis, and the mycobacterial culture provides microbiologic confirmation. Drug susceptibility testing can be used to guide anti-TB therapy [10,11].

The treatment for peritoneal TB is mainly pharmacologic and involves using the same six-month regimen that is used for the treatment of pulmonary TB. Isoniazid (INH), rifampin (RIF), ethambutol, pyrazinamide, and streptomycin are commonly used first-line medications. A four-drug regimen is administered for two months, followed by the continuation of treatment with isoniazid and rifampin for four or more months. However, patients with preexisting liver disease may be at an increased risk of developing hepatotoxicity with the use of isoniazid, rifampin, or pyrazinamide. In such patients, a closer monitoring of the liver function or even alternative regimens with lower hepatotoxic potential may be considered. The response to the anti-TB treatment is usually noted within the first three months and is manifested by the resolution of presenting symptoms and the normalization of laboratory values. Surgical intervention is reserved for patients who develop intestinal perforation, bowel obstruction, abscesses, fistulae, or hemorrhage [12-15].

Peritoneal TB is a rare condition in Western countries; however, it should be suspected in patients who are immigrants or have other high-risk factors. Our patient who emigrated to the United States from Bangladesh, a country with a high TB burden [16,17], was immunocompetent and had no other known risk factors for developing peritoneal TB. Laparoscopy and peritoneal biopsy aided in establishing the diagnosis, and the patient was started on anti-TB therapy. Significant clinical improvement was observed in two months. This case highlights that the clinicians must be aware of the clinical characteristics in the immigrant population and maintain a high index of suspicion for peritoneal TB as a cause of new-onset ascites in immunocompetent patients with no other high-risk factors. Prompt diagnosis and treatment can lead to the resolution of symptoms, whereas delay in the initiation of therapy may be associated with significant mortality [3].

## Conclusions

Peritoneal TB is an uncommon medical condition with a higher chance of occurring in association with immunocompromised states. However, it may rarely develop in the absence of immunosuppression, particularly in immigrants belonging to TB-endemic areas. Therefore, a high degree of suspicion should be maintained to diagnose this condition in patients presenting with new-onset lymphocytic ascites. The identification of peritoneal TB may be challenging because of the presence of nonspecific clinical manifestations and the limited yield of most non-invasive diagnostic tests. Laparoscopy with peritoneal biopsy remains the gold standard diagnostic investigation and treatment is with the same anti-TB treatment regimen used for the management of pulmonary TB.
